# Effect of Maternal Table Tilt During Cesarean Delivery Under Spinal Anesthesia on Norepinephrine Requirements: A Prospective Observational Comparative Study

**DOI:** 10.3390/healthcare14010117

**Published:** 2026-01-03

**Authors:** Jakub Vallo, Jana Morávková, Matúš Paulíny, Peter Sabaka

**Affiliations:** 1 Department of Anestesiology, Slovak Medical University, 833 05 Bratislava, Slovakia; jakub.vallo@gmail.com (J.V.); morvakova.jana@gmail.com (J.M.); matus.pauliny@kr.unb.sk (M.P.); 2 Department of Anestesiology, University Hospital in Bratislava, 821 01 Bratislava, Slovakia; 3 Department of Infectology and Geographical Medicine, Faculty of Medicine, Comenius University in Bratislava, 833 05 Bratislava, Slovakia

**Keywords:** cesarean section, spinal anesthesia, norepinephrine, tilt, aortocaval compression

## Abstract

**Background:** Left lateral tilt is traditionally recommended during cesarean delivery to reduce aortocaval compression and maintain maternal hemodynamic stability; however, with the widespread adoption of prophylactic vasopressor strategies recommended by current guidelines, the incremental benefit of routine tilt remains uncertain. **Methods:** We conducted a prospective, nonrandomized observational comparative study at the University Hospital Bratislava including 99 women undergoing elective cesarean delivery under spinal anesthesia. Participants were managed either with a standard ~15° left lateral tilt (*n* = 41) or in a flat supine position without tilt (*n* = 58), according to the day of surgery and routine anesthesiologist practice; all other anesthetic and surgical procedures were identical. A prophylactic norepinephrine infusion was initiated at 0.05 µg/kg/min and titrated to maintain systolic arterial pressure at 90–100% of baseline. The primary outcome was the average norepinephrine infusion rate (µg/kg/min) from induction of spinal anesthesia to neonatal delivery. Secondary outcomes included total norepinephrine dose to delivery, dose normalized per kilogram, and neonatal outcomes (Apgar scores and umbilical arterial blood gas parameters). **Results:** The median norepinephrine infusion rate was 0.03 µg/kg/min in both groups (tilt: IQR 0.01–0.04 vs. no-tilt: IQR 0.02–0.04; *p* = 0.325). Total norepinephrine dose to delivery (20 [15–35] µg; *p* = 0.89) and dose per kilogram (0.25 [0.15–0.33] µg/kg vs. 0.34 [0.17–0.44] µg/kg; *p* = 0.10) were also comparable. Neonatal outcomes, including Apgar scores and umbilical arterial blood gas parameters, did not differ significantly between groups. In a multivariable regression sensitivity analysis adjusting for maternal and procedural covariates, table tilt was not independently associated with norepinephrine requirements. **Conclusions:** In parturients undergoing cesarean delivery under spinal anesthesia with prophylactic norepinephrine infusion, a 15° left lateral tilt did not reduce vasopressor requirements or improve neonatal outcomes. Routine maternal tilt therefore appears unnecessary for hemodynamic optimization in this setting, and patient positioning can be individualized without compromising maternal or neonatal safety.

## 1. Introduction

Spinal anesthesia is widely preferred for cesarean delivery due to its rapid onset, dense block, minimal maternal–fetal drug exposure, and high satisfaction rates [1,2,3]. However, a well-recognized drawback is spinal-induced hypotension, historically reported in up to 70–80% of cases without prophylaxis [4,5]. The principal mechanism is sympathetic blockade, which leads to reduced systemic vascular resistance, venous pooling, decreased venous return, and—if uncorrected—compromised uteroplacental perfusion with potential fetal acidosis [6,7,8]. To counteract mechanical aortocaval compression by the gravid uterus in the supine position, obstetric anesthesia practice traditionally applies left lateral tilt—commonly ~15°—to displace the uterus off the inferior vena cava and aorta [9,10]. This recommendation emerged in an era when vasopressors were administered reactively (often ephedrine), fluids were used liberally, and continuous vasopressor infusions were uncommon [7,8,9]. Over the past decade, clinical practice has shifted toward the use of prophylactic vasopressor infusions during spinal anesthesia for cesarean delivery, supported by international consensus statements and narrative reviews [2,3]. Phenylephrine effectively maintains arterial pressure but may reduce heart rate and cardiac output, whereas norepinephrine—owing to its mixed α-adrenergic and modest β-adrenergic activity—better preserves heart rate and cardiac output while providing comparable blood pressure control [11,12,13,14,15]. In this contemporary setting, the incremental hemodynamic benefit of routine maternal table tilt has increasingly been questioned. Both hemodynamic investigations and randomized studies have reported similar cardiac output and arterial pressure in supine versus tilted positions when continuous vasopressor infusions are employed [12,13,14,15,16]. Moreover, excessive tilt may impair surgical exposure and influence the cephalad spread and distribution of hyperbaric local anesthetics [17]. Nevertheless, the routine use of left lateral tilt in modern obstetric anesthesia practice remains a subject of ongoing debate. We therefore evaluated whether maintaining a standard left lateral tilt during cesarean delivery under spinal anesthesia reduces norepinephrine requirements compared with a flat supine position when a standardized prophylactic norepinephrine infusion protocol is used. We hypothesized that maternal table tilt would not meaningfully affect vasopressor requirements or neonatal outcomes. This study aims to provide detailed methodological information to facilitate reproducibility and to contextualize the findings within current evidence and clinical guidelines.

## 2. Methods

### 2.1. Study Design and Setting

We conducted a prospective, single-center, nonrandomized, observational comparative study to evaluate the effect of left lateral tilt during spinal anesthesia for cesarean delivery on vasopressor requirements. The study was carried out at the Department of Gynecology, University Hospital Bratislava, between 1 December 2021 and 30 June 2022. Two institutional approaches to patient positioning during elective cesarean delivery under spinal anesthesia were compared: an approximately 15° left lateral tilt and a flat supine position. The study protocol was approved by the Institutional Ethics Committee of the University Hospital Bratislava (ECK122020; 21 March 2020). The study was conducted in accordance with the Declaration of Helsinki and the principles of Good Clinical Practice. Written informed consent was obtained from all participants prior to enrollment. No external funding was received for this study.

### 2.2. Participants and Eligibility Criteria

Adult parturients scheduled for cesarean delivery at the study institution were consecutively enrolled. Inclusion criteria were adult women (≥18 years) with ASA physical status II, singleton term pregnancy (≥37 weeks), and a scheduled elective cesarean delivery under spinal anesthesia. All participants were hemodynamically stable prior to anesthesia and free of major obstetric or systemic diseases that could confound hemodynamic assessment. Exclusion criteria included conversion to general anesthesia; emergency or urgent cesarean delivery; preeclampsia or chronic hypertension; known cardiovascular disease (ischemic heart disease, valvular disease, or cardiomyopathy); multiple gestation; intraoperative hemorrhage unrelated to spinal anesthesia; or use of vasopressors for indications other than spinal anesthesia–related hypotension (e.g., sepsis or anaphylaxis). Baseline demographic and obstetric data (age, height, weight, BMI, gestational age, and fetal weight) were collected prospectively using a standardized case report form and cross-checked with electronic medical records.

### 2.3. Group Allocation and Blinding

Participants were allocated according to the day of surgery reflecting routine practice of the attending anesthesiologist: those operated on odd calendar days received approximately 15° left lateral tilt; those on even days were managed in a flat supine position. Allocation to positioning groups was based on calendar day and anesthesiologist workflow and did not involve random assignment. This pragmatic allocation reflected real-world workflow while minimizing subjective selection bias. The anesthesiologist and surgical team were aware of the position, which was unavoidable; however, the investigator responsible for data capture, data entry, and statistical analysis was blinded to the group assignment. Group codes were revealed only after all data had been analyzed.

### 2.4. Anesthetic Technique and Standardization of Care

All procedures were performed in a temperature-controlled operating room (22–24 °C). Standard monitoring included electrocardiography, pulse oximetry, and noninvasive blood pressure (NIBP) measurement using a calibrated automated monitor (Infinity Delta; Drägerwerk AG, Lübeck, Germany). Appropriate cuff sizes were selected based on arm circumference. Intravenous access was established using an 18-gauge cannula. Spinal anesthesia was performed in the sitting position using a 25-gauge pencil-point needle at the L3–L4 or L4–L5 interspace. After confirmation of free cerebrospinal fluid flow, the following anesthetic mixture was injected intrathecally over 10–15 s: 12 mg of 0.5% hyperbaric bupivacaine, 10 µg fentanyl, and 3 µg sufentanyl. Immediately after injection, patients were positioned according to group allocation (left lateral tilt or flat supine position). Oxygen at 2 L/min was administered via nasal cannula until delivery. A crystalloid co-load (Ringer’s lactate, 5–7 mL/kg) was infused within the first 10 min after spinal injection. Adequacy of sensory block was confirmed at the T4–T6 dermatomal level prior to skin incision.

### 2.5. Positioning and Verification of Tilt

In the tilt group, a rigid wedge was placed under the right buttock and flank to achieve a table tilt of approximately 15°. The angle was verified before each procedure using a digital inclinometer placed on the operating table surface. The wedge remained in place until neonatal delivery, after which patients were returned to the supine position. In the no-tilt group, the operating table remained flat throughout the procedure. Aside from patient positioning, anesthetic and surgical management were identical in both groups.

### 2.6. Vasopressor Protocol

Immediately after spinal anesthesia, a prophylactic norepinephrine infusion was initiated at 0.05 µg/kg/min (norepinephrine 4 mg diluted in 500 mL of 5% glucose solution, resulting in a concentration of 8 µg/mL) using a calibrated syringe pump (Perfusor^®^ Compact, B. Braun Melsungen AG, Hessen, Germany). The infusion rate was adjusted every 30 s to maintain systolic arterial pressure (SAP) between 90% and 100% of baseline, defined as the mean of three pre-spinal measurements obtained in the semi-recumbent position with left uterine displacement. If SAP decreased to below 80% of baseline or <90 mmHg, a 10 µg intravenous norepinephrine bolus was administered and the infusion rate was increased. Bradycardia (heart rate <50 bpm accompanied by hypotension) was treated with atropine 0.5 mg intravenously. Nausea or vomiting were treated with ondansetron 4 mg intravenously as required. All norepinephrine adjustments, including changes in infusion rate and bolus administration, were automatically recorded by the infusion pump and cross-checked against the anesthesia record to ensure accuracy and traceability.

### 2.7. Outcomes and Data Collection

The primary outcome was the average norepinephrine infusion rate (µg/kg/min) from induction of spinal anesthesia to neonatal delivery. The average infusion rate was calculated as a time-weighted mean based on infusion pump logs, with each rate setting weighted by the duration for which it was maintained, including all adjustments performed at 30 s intervals. Secondary outcomes included: (i) total norepinephrine dose to delivery (µg), (ii) total norepinephrine dose normalized per kilogram body weight (µg/kg), (iii) time from spinal anesthesia to delivery, (iv) time from skin incision to delivery, and (v) neonatal outcomes, including Apgar scores at 1 and 5 min and umbilical arterial blood gas parameters (pH, base excess, HCO_3_^−^, pCO_2_, pO_2_, oxygen saturation, and ctCO_2_). Hemodynamic variables (systolic and mean arterial pressure, heart rate, and oxygen saturation) were recorded at 1 min intervals until delivery and at 3–5 min intervals thereafter. Umbilical arterial blood samples were obtained immediately after cord clamping using pre-heparinized syringes and analyzed within 5 min using a blood gas analyzer (ABL800 FLEX, Radiometer, Denmark). Data were entered into a standardized case report form by a trained investigator blinded to group allocation. Data completeness was verified daily; missing data accounted for <1% of observations and did not affect the primary endpoint, and therefore no data imputation was performed.

### 2.8. Statistical Analysis

All analyses were conducted using IBM SPSS Statistics, version 29 (IBM Corp., Armonk, NY, USA). Continuous variables were expressed as median with interquartile range (IQR) and compared using the Mann–Whitney U test because of non-normal distribution, as confirmed by the Shapiro–Wilk test. Categorical variables were analyzed using the χ^2^ test or Fisher’s exact test, as appropriate. Effect sizes were calculated as Hedges’ g and Cliff’s δ, each with 95% confidence intervals (CIs). The Hodges–Lehmann estimator was used to calculate median differences with corresponding CIs. A post hoc descriptive analysis based on effect size estimates and their corresponding confidence intervals was performed to assess the magnitude and precision of the between-group difference. To assess robustness against potential confounding arising from the nonrandomized allocation, a prespecified multivariable regression sensitivity analysis was conducted with average norepinephrine infusion rate (µg/kg/min) as the dependent variable, adjusting for maternal BMI, maternal age, gestational age, and time from spinal anesthesia to delivery. All statistical tests were two-tailed, and a *p* value < 0.05 was considered statistically significant.

## 3. Results

### 3.1. Participants

**Figure 1:**
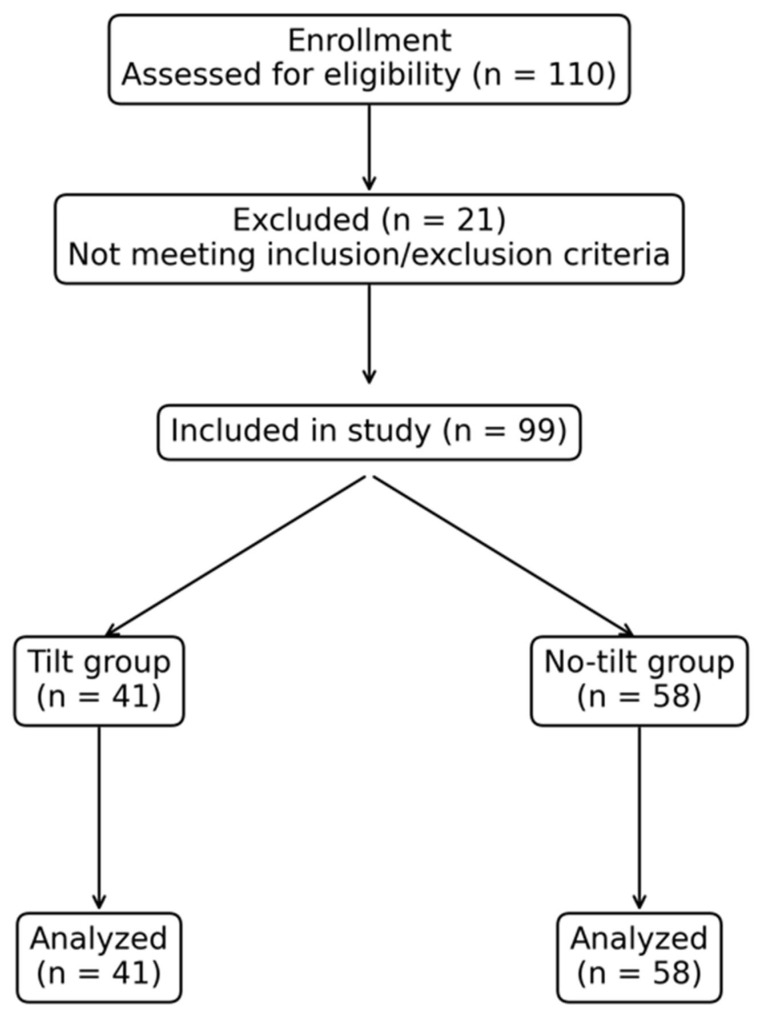
CONSORT flow diagram of patient enrollment and analysis.

A total of 99 patients were included in the study: 41 in the tilt group and 58 in the no-tilt group. Initially, 110 patients were screened; 21 were excluded prior to study inclusion because they did not meet the inclusion or exclusion criteria (Figure 1). Baseline maternal characteristics (age, height, weight) and obstetric variables were comparable between the two groups (Table 1). There were no protocol deviations, conversions to general anesthesia, or exclusions after enrollment.

### 3.2. Maternal Outcomes

The median norepinephrine infusion rate was 0.03 µg/kg/min in both groups (tilt: IQR 0.01–0.04; no-tilt: IQR 0.02–0.04; *p* = 0.325). Total norepinephrine dose to delivery was 20 µg (IQR 15–30) in the tilt group and 25 µg (IQR 15–35) in the no-tilt group (*p* = 0.89). The total dose normalized per kilogram body weight did not differ between groups (0.25 [0.15–0.33] Vs. 0.34 [0.17–0.44] µg/kg; *p* = 0.10). Time from spinal anesthesia to delivery and time from skin incision to delivery were similar in both groups. Two maternal adverse events were recorded. Mild nausea requiring treatment with ondansetron occurred in two patients in the tilt group. No episodes of bradycardia were observed in either group.

### 3.3. Neonatal Outcomes

Apgar scores at 1 and 5 min were ≥9 in both groups. Umbilical arterial blood gas parameters, including pH, base excess, HCO_3_^−^, pCO_2_, pO_2_, oxygen saturation, and ctCO_2_, did not differ significantly between groups (Table 2). No neonate required advanced resuscitation or admission to the neonatal intensive care unit for hemodynamic compromise.

### 3.4. Post Hoc Power Analysis

The standardized between-group difference for the primary outcome was very small (Hedges’ g = 0.13; Cliff’s δ = 0.07). The Hodges–Lehmann median difference (no-tilt minus tilt) was 0.002 µg/kg/min (95% CI −0.004 to 0.008). The narrow confidence interval excludes moderate or large differences between groups but does not exclude very small effects with potential clinical relevance. These findings indicate substantial overlap in norepinephrine requirements between positions.

### 3.5. Multivariate Analysis

To evaluate robustness of the primary findings against potential confounding introduced by the nonrandomized allocation, a prespecified multivariable regression sensitivity analysis was performed. Average norepinephrine infusion rate was used as the dependent variable, with group allocation (tilt vs. no-tilt) and clinically relevant covariates included in the model. After adjustment for maternal BMI, maternal age, gestational age, and time from spinal anesthesia to delivery, table tilt was not independently associated with norepinephrine requirements (adjusted β = −0.00475 µg/kg/min; 95% CI −0.01241 to 0.00292; *p* = 0.225) (Table 3). The adjusted effect size and direction were consistent with the unadjusted analysis.

## 4. Discussion

In this prospective observational study, routine application of an approximately 15° left lateral tilt during cesarean delivery under spinal anesthesia did not reduce norepinephrine requirements or improve neonatal outcomes compared with a flat supine position when a standardized prophylactic norepinephrine infusion protocol was used. These findings reinforce growing evidence that, in the era of continuous vasopressor infusion, mechanical left uterine displacement adds little hemodynamic benefit when maternal blood pressure and cardiac output are pharmacologically maintained.

### 4.1. Comparison with Previous Studies

Our results are consistent with several recent randomized and crossover studies demonstrating that maternal tilt does not significantly alter cardiac output, stroke volume, or systolic arterial pressure when active vasopressor management is implemented. Dyer et al. reported negligible differences in hemodynamic parameters between 0° and 15° tilt during spinal anesthesia for cesarean delivery when a phenylephrine infusion was used to maintain blood pressure [12]. Similarly, Onwochei et al. found that left lateral tilt did not meaningfully prevent aortocaval compression as assessed by magnetic resonance imaging [13]. Park et al. confirmed comparable blood pressure and cardiac output between 0° and 15° tilt in parturients receiving prophylactic phenylephrine infusion [14]. Our data extend these observations to norepinephrine, which is increasingly preferred over phenylephrine because of its more favorable hemodynamic profile and lower incidence of reflex bradycardia [11,15,18]. Studies directly comparing norepinephrine and phenylephrine have shown that norepinephrine more effectively preserves heart rate and cardiac output while providing equivalent blood pressure control [11,18,19]. Consequently, under continuous norepinephrine infusion, mechanical maternal tilt may no longer confer a measurable additional hemodynamic benefit. In contrast, earlier studies supporting routine tilt were conducted in a markedly different hemodynamic context, characterized by reactive ephedrine boluses and liberal fluid administration [7,8,9]. For example, Corke et al. reported a higher incidence of hypotension in the supine position; however, this finding occurred in the absence of prophylactic vasopressor use [8]. Our findings suggest that when blood pressure is proactively maintained within 90–100% of baseline using a norepinephrine infusion, the hemodynamic consequences of aortocaval compression are effectively compensated. Recent meta-analyses further support this paradigm shift. A systematic review of maternal positioning during cesarean section under spinal anesthesia concluded that there is insufficient evidence that lateral tilt improves maternal hemodynamics or neonatal acid–base status when vasopressor prophylaxis is applied [20]. Similarly, Guo et al. (2023) reported that 15° or 20° tilt had no significant effect on umbilical arterial pH, Apgar scores, or vasopressor dose requirements compared with the supine position [21]. In addition, a mechanistic study using transthoracic echocardiography demonstrated only minimal changes in inferior vena cava collapsibility and cardiac output with 15° tilt, indicating that mechanical decompression of the great vessels is incomplete and of limited relevance under contemporary anesthetic conditions [22].

### 4.2. Interpretation and Clinical Implications

Physiologically, norepinephrine counteracts the vasodilatory effects of sympathetic blockade through α-adrenergic–mediated vasoconstriction while preserving cardiac output via modest β_1_-adrenergic stimulation. In this controlled hemodynamic environment, the incremental contribution of maternal tilt to preload preservation becomes negligible. The comparable norepinephrine infusion rates and neonatal blood gas parameters observed in both groups in our study reinforce the concept that pharmacologic stabilization supersedes mechanical compensation. From a practical perspective, omitting routine tilt may improve surgical ergonomics, facilitate patient positioning and surgical exposure, and reduce the risk of uneven cephalad spread of hyperbaric bupivacaine [17]. It may also simplify intraoperative workflow and shorten operating room setup time without compromising maternal or neonatal safety. Accordingly, an individualized approach to maternal positioning—reserved for specific clinical scenarios such as morbid obesity, multiple gestation, or intraoperative uterine manipulation—appears to be a rational, evidence-based strategy [16].

### 4.3. Original Contribution of the Present Study

This study adds several important elements to the existing body of knowledge. First, it is one of the few prospective analyses focusing on norepinephrine infusion in the context of maternal positioning, providing real-world data derived from a standardized and reproducible institutional protocol. Second, blinded data collection and detailed vasopressor titration records strengthen the internal validity and reproducibility of our findings. Third, by incorporating post hoc sensitivity and power analyses, this work quantifies the minimal detectable effect size, offering a level of methodological transparency that is rarely reported in obstetric anesthesia research. Finally, by demonstrating hemodynamic equivalence between positioning strategies in a well-controlled cohort, our results support the concept that routine mechanical uterine displacement may be unnecessary when continuous vasopressor prophylaxis is employed.

### 4.4. Limitations and Future Directions

This study has limitations inherent to its observational design. Group allocation was nonrandomized, and subtle differences in operator technique cannot be entirely excluded. Nevertheless, restriction of enrollment to ASA physical status II parturients reduced baseline clinical heterogeneity. Importantly, sensitivity analyses adjusting for available maternal and procedural covariates yielded results consistent with the unadjusted comparisons, indicating that the absence of an association between table tilt and norepinephrine requirements is unlikely to be explained by measured confounding. Although the study was sufficiently precise to exclude moderate or large differences in norepinephrine requirements, very small effects cannot be definitively ruled out, as no prespecified equivalence or noninferiority margin was defined a priori. Also, the pragmatic, nonrandomized allocation led to an imbalance in group sizes between the tilt and no-tilt groups, which may introduce residual confounding despite adjustment in prespecified sensitivity analyses.

Furthermore, the study population included only healthy term parturients undergoing elective cesarean delivery; therefore, extrapolation of these findings to higher-risk populations (e.g., severe preeclampsia, multiple gestations, or morbid obesity) should be undertaken with caution. Future randomized multicenter trials incorporating continuous cardiac output monitoring and assessment of uteroplacental perfusion may further delineate subgroups that could still benefit from partial lateral positioning [21,22]. In addition, evaluation of workflow efficiency, anesthetic spread dynamics, and patient comfort could help define the broader clinical and logistical impact of abandoning routine maternal tilt.

It should also be acknowledged that colloid solutions may provide more effective intravascular volume expansion than crystalloids and have been associated with a lower incidence of spinal anesthesia–induced hypotension in earlier studies. Their role may therefore be more relevant in clinical settings where vasopressor use is constrained or unavailable. However, given concerns regarding cost, availability, and potential adverse effects, together with the widespread adoption of prophylactic vasopressor infusions, colloids are no longer routinely recommended for this purpose [2,3].

Finally, because the analysis focused on the interval from spinal anesthesia to delivery and excluded major hemorrhagic events, surgical variables such as estimated blood loss and transfusion requirements were not systematically captured.

## 5. Conclusions

In parturients undergoing cesarean delivery under spinal anesthesia with prophylactic norepinephrine infusion, we did not observe a reduction in vasopressor requirements or neonatal outcomes associated with routine 15° left lateral tilt, although small effects cannot be excluded.

## Figures and Tables

**Table 1:** Summary of maternal baseline variables and outcomes.

Variable	Tilt Median (IQR)	No Tilt Median (IQR)	*p*-Value
Norepinephrine total dose (µg)	20.00 (15.00–30.00)	25.00 (15.00–35.00)	0.8947
Age	34.00 (31.00–37.00)	35.50 (32.00–38.00)	0.5495
Weight (kg)	80.00 (72.00–90.00)	78.00 (70.25–89.00)	0.5866
Height (cm)	168.00 (164.00–172.00)	168.00 (162.50–170.00)	0.5592
Time from SAB to delivery (min)	10.00 (9.00–11.00)	10.00 (9.00–11.00)	0.5773
Time from incision to delivery (min)	5.00 (4.00–5.00)	5.00 (4.00–5.00)	0.6024
Norepinephrine dosing (µg/kg/min)	0.03 (0.01–0.04)	0.03 (0.02–0.04)	0.3250
Norepinephrine total dose per kg (µg/kg)	0.25 (0.15–0.33)	0.34 (0.17–0.44)	0.1008

Values are presented as median (interquartile range). Abbreviations: SAB—subarachnoid block; µg—microgram; min—minute; kg—kilogram; IQR—interquartile range.

**Table 2:** Summary of neonatal baseline variables and outcomes.

	Tilt Median (IQR)	No Tilt Median (IQR)	*p*-Value
Gestational age (weeks)	39.00 (38.00–39.00)	39.00 (38.00–40.00)	0.8795
Newborn length (cm)	50.00 (48.00–51.00)	49.00 (48.00–50.00)	0.1153
Newborn weight (g)	3260.00 (2950.00–3740.00)	3305.00 (2995.00–3582.50)	0.6908
apgar 1	10.00 (9.00–10.00)	10.00 (9.00–10.00)	0.7281
apgar 5	10.00 (10.00–10.00)	10.00 (10.00–10.00)	0.5244
pH	7.35 (7.31–7.36)	7.33 (7.31–7.35)	0.1053
BB (mmol/L)	45.30 (44.10–46.40)	45.15 (44.02–46.33)	0.6698
BE (mmol/L)	−2.40 (−3.60–−2.00)	−2.95 (−4.07–−2.15)	0.3078
HCO_3_^−^ (mmol/L)	22.80 (21.20–23.90)	23.15 (21.90–24.15)	0.2661
pCO_2_ (kPa)	5.77 (5.30–6.26)	5.88 (5.52–6.61)	0.1123
pO_2_ (kPa)	3.70 (3.00–4.50)	3.45 (2.90–4.40)	0.5914
satO_2_	0.60 (0.43–0.72)	0.56 (0.41–0.73)	0.6855
ctCO_2_	24.01 (22.31–25.28)	24.23 (23.12–25.47)	0.4731

Values are presented as median (interquartile range). Abbreviations: BE—base excess; BB—buffer base; HCO_3_^−^—bicarbonate; kPa—kilopascal; mmol/L—millimoll per liter; pCO_2_—partial pressure of carbon dioxide; pO_2_—partial pressure of oxygen; satO_2_—oxygen saturation; ctCO_2_—total carbon dioxide content; cm—centimeter; g—gram; IQR—interquartile range.

**Table 3:** Multivariable regression analysis of average norepinephrine infusion rate.

Variable	Adjusted β (µg/kg/min)	95% CI	*p*-Value
Table tilt (vs no-tilt)	−0.00475	−0.01241–0.00292	0.225
Maternal BMI (kg/m^2^)	0.00021	−0.00015–0.00057	0.247
Maternal age (years)	−0.00009	−0.00042–0.00024	0.586
Gestational age (weeks)	0.00031	−0.00088–0.00150	0.611
Time from SAB to delivery (min)	0.00058	−0.00034–0.00150	0.215

Values are presented as adjusted β coefficients with 95% confidence intervals from a multivariable linear regression model with heteroscedasticity-robust standard errors (HC3). The dependent variable was average norepinephrine infusion rate (µg/kg/min). Sample size: *n* = 96 (complete cases). Abbreviations: BMI—body mass index; CI—confidence interval; SAB—subarachnoid block.

## Data Availability

The anonymized raw dataset supporting this study is included in Supplementary Materials.

## Supplementary Materials

The following supporting information can be downloaded at: https://www.mdpi.com/article/10.3390/healthcare14010117/s1. File S1: Supplementary Data.

## Abbreviations

The following abbreviations are used in this manuscript:

ASA	American Society of Anesthesiologists
BE	Base Excess
BB	Buffer Base
BMI	Body Mass Index
BP	Blood Pressure
CI	Confidence Interval
Cm	Centimeter
CO	Cardiac Output
ctCO_2_	Total Carbon Dioxide Content
G	Gram
HL	Hodges–Lehmann
HCO_3_^−^	Bicarbonate
HR	Heart Rate
IQR	Interquartile Range
MCID	Minimal Clinically Important Difference
MDES	Minimal Detectable Effect Size
Min	Minute
NA	Norepinephrine
NE	Norepinephrine
pCO_2_	Partial Pressure of Carbon Dioxide
pO_2_	Partial Pressure of Oxygen
SAB	Subarachnoid Block
satO_2_	Oxygen Saturation
SD	Standard Deviation
SPSS	Statistical Package for the Social Sciences
µg	Microgram
µg/kg/min	Microgram per kilogram per minute
A	Alpha
Δ	Delta
g (Hedges’)	Standardized Mean Difference
